# An integrative Bayesian Dirichlet-multinomial regression model for the analysis of taxonomic abundances in microbiome data

**DOI:** 10.1186/s12859-017-1516-0

**Published:** 2017-02-08

**Authors:** W. Duncan Wadsworth, Raffaele Argiento, Michele Guindani, Jessica Galloway-Pena, Samuel A. Shelbourne, Marina Vannucci

**Affiliations:** 1 Department of Statistics, Rice University, Houston, TX USA; 2 ESOMAS Department, University of Torino and Collegio Carlo Alberto, Torino, Italy; 3 Department of Statistics, University of California, Irvine, CA USA; 4 Department of Infectious Disease, Infection Control, and Employee Health, The University of Texas MD Anderson Cancer Center, Houston, 77030 TX USA; 5 Department of Genomic Medicine, The University of Texas MD Anderson Cancer Center, Houston, 77030 TX USA

**Keywords:** Bayesian hierarchical model, Data integration, Dirichlet-multinomial, Microbiome data, Variable selection

## Abstract

**Background:**

The Human Microbiome has been variously associated with the immune-regulatory mechanisms involved in the prevention or development of many non-infectious human diseases such as autoimmunity, allergy and cancer. Integrative approaches which aim at associating the composition of the human microbiome with other available information, such as clinical covariates and environmental predictors, are paramount to develop a more complete understanding of the role of microbiome in disease development.

**Results:**

In this manuscript, we propose a Bayesian Dirichlet-Multinomial regression model which uses *spike-and-slab* priors for the selection of significant associations between a set of available covariates and taxa from a microbiome abundance table. The approach allows straightforward incorporation of the covariates through a log-linear regression parametrization of the parameters of the Dirichlet-Multinomial likelihood. Inference is conducted through a Markov Chain Monte Carlo algorithm, and selection of the significant covariates is based upon the assessment of posterior probabilities of inclusions and the thresholding of the Bayesian false discovery rate. We design a simulation study to evaluate the performance of the proposed method, and then apply our model on a publicly available dataset obtained from the Human Microbiome Project which associates taxa abundances with KEGG orthology pathways. The method is implemented in specifically developed R code, which has been made publicly available.

**Conclusions:**

Our method compares favorably in simulations to several recently proposed approaches for similarly structured data, in terms of increased accuracy and reduced false positive as well as false negative rates. In the application to the data from the Human Microbiome Project, a close evaluation of the biological significance of our findings confirms existing associations in the literature.

**Electronic supplementary material:**

The online version of this article (doi:10.1186/s12859-017-1516-0) contains supplementary material, which is available to authorized users.

## Background

The human microbiome is defined as the collection of microorganisms, including bacteria, viruses, and some unicellular eukaryotes, that live in and on our bodies [1]. Research on the microbiome has grown exponentially in the past few years and it has been argued that the microbiota can be regarded as a “second genome”[2, 3]. Indeed, just the human gut microbiome is estimated to be composed of approximately 10^14^ bacterial cells, i.e. ten times more than the total number of human cells in the body [4]. The contribution of the human microbiome on several health outcomes has been frequently reported in the literature. For example, microbial dysbiosis in the gut has been linked to irritable bowel syndrome and Crohn’s disease [5], type 2 diabetes [6], cardiovascular disease [7], and psychological conditions via the so-called “gut-brain axis” [8]. The composition of microbiota at other body sites have also been associated with conditions such as eczema [9] and pre-term labor [10]. This stream of research holds great potential for a better understanding of many mechanistic processes in the development of human diseases, especially with respect to immune regulation and barrier defense [11, 12].

Microbiome data is most commonly obtained by sequencing variable regions of the 16S rRNA gene, then grouping the transcripts into Operational Taxonomic Units (OTUs), based on their similarity to one another. The OTUs are then defined as a cluster of reads based on a similarity threshold (typically, 97%) set by the researcher. The membership count of each cluster is then used as a proxy for taxa abundances in the sample [13]. See [14] for a discussion of how the selection of the cutoff might impact the resulting OTUs, in particular for rare species. Many studies summarize the taxa abundances by constructing several indicators of community composition (e.g. alpha and beta diversity indexes, see, [15]). Alternatively, the full OTUs abundance table can be used to obtain more detailed information about existing associations between environment or phenotypes and microbes. Well-established statistical models for the analysis of count data (e.g., Poisson or Negative Binomial distribution) can be efficaciously employed for the analysis of taxonomic count data [16]. Although less common, other distributions (e.g., a two-parameter Weibull distribution) have also been shown to provide a good fit to the data for some communities (see, e.g. [17]). One distinctive characteristic of the microbiome data is their overdispersion: while some taxa (e.g., *Bacteroides* and *Lactobacillus* species) are common among samples, many other taxa are present at much lower abundances, and often never recorded in a sample, leading to zero-inflated distributions. Many of the existing tools for microbial community analysis (e.g., the QIIME platform, [18]) bypass those characteristics and rely on nonparametric tests to compare species across different conditions [19, 20]. Other approaches use ordination, e.g. multidimensional scaling, to summarize abundances, and are sometimes employed to link the microbiome data with available clinical covariates and phylogenetic information [21, 22]. In those approaches, the choice of the distance metric is often crucial. The interpretation of biological phenomena can also be challenging in low dimensional projections. Most importantly, distance-based methods do not explicitly quantify the relative importance of significant associations between taxa and covariates, and therefore are of limited use for clinical decisions.

In this manuscript, we consider an integrative Bayesian approach based on the use of Dirichlet-Multinomial (DM) distributions [23] for studying the association between taxa abundance data and available measurements on clinical, genetic and environmental covariates. Recently, La Rosa et al. [24] proposed the use of a DM model for hypothesis testing and power calculations in microbiome experiments. Holmes et. al [25] used a finite mixture of DM distributions to directly model the taxa counts. Neither method incorporate predictors to study the influence of external factors on the microbiome’s abundance. A penalized likelihood approach based on a DM regression model has been proposed instead by [26] to determine significant associations between the microbiome composition and a set of covariates which describe the individual dietary nutrients’ intakes. Similarly, [27] develop a structure constrained version of sparse canonical correlation analysis that integrates compositionalized microbiome data, phylogenetic information, and nutrient information. Furthermore, [28] propose penalized regression models to associate the multivariate compositionalized microbiome data with some univariate phenotype of interest, e.g. body mass index, as a response. However, the use of a constrained optimization approach does not allow to fully characterize the uncertainty in the selection of the significant associations, which is of particular importance, especially when dealing with high-dimensional and highly-correlated data.

Here, we propose a probabilistic modeling approach which both flexibly takes into account the typical features of microbiome count data and also allows for straightforward incorporation of available covariate information within a DM log-linear regression framework. With respect to modeling approaches as in [28], our framework allows the study of associations between multivariate microbiome data and multivariable predictors. By imposing sparsity inducing *spike-and-slab priors* on the regression coefficients, our model obtains a parsimonious summary of the effects of the associations and also allows an assessment of the uncertainty of the selection process. We evaluate the performance of our model first on simulated data, where we provide comparisons with methods developed for microbiome or similar type of data. We also illustrate our method on data obtained from the Human Microbiome Project [29], to investigate the association between taxonomic abundances and metabolic pathways inferred from whole genome shotgun sequencing reads. It is known that the combination of environmental and host genetic factors shape the composition of the gut microbiota, and these interactions appear to have a significant effect on several biological mechanisms, which may be related, for example, to the individual immunity and barrier defense, as well as metabolism and diet [30, 31]. The approach has been implemented in a user-friendly R code, which has been made publicly available (see the Licensing Section).

## Methods

We describe our Bayesian variable selection approach for the analysis of microbiome data and their association with a set of available covariates in the context of DM log-linear regression models.

### Dirichlet-multinomial regression with variable selection

Let **y**_*i*_=(*y*_*i*1_,…,*y*_*iJ*_) indicate the vector of counts representing the taxonomic abundance table obtained from the *i*th patient, with *y*_*ij*_ denoting the frequency of the *j*th microbial taxon, for *j*=1,…,*J* and *i*=1,…,*n*. Furthermore, let **X**=(**x**_1_,…,**x**_*P*_) indicate a *n*×*P* matrix of measurements on *P* covariates. We start by modeling the taxonomic count data with a Multinomial distribution

1 $$\begin{array}{@{}rcl@{}} \boldsymbol{y}_{i} \mid \boldsymbol{\phi}_{i} \sim \text{Multinomial}\left(y_{i+}, \boldsymbol{\phi}_{i}\right),  \end{array} $$

with $y_{i+}=\sum _{j=1}^{J} y_{ij}$ the summation of all counts in the vector, and where the parameters ***ϕ***’s are defined on the *J* dimensional simplex

$$\begin{array}{@{}rcl@{}} \mathcal{S}^{J-1} = \left\{ \left(\phi_{1}, \dots, \phi_{J}\right) : \phi_{j} \ge 0, \; \forall j, \; \textstyle \sum_{j = 1}^{J} \phi_{j} = 1 \right\}, \end{array} $$

We further impose a conjugate Dirichlet prior on ***ϕ***, that is ***ϕ***∼Dirichlet(***γ***), where ***γ***=(*γ*_1_,…,*γ*_*J*_) indicates a *J*-dimensional vector of strictly positive parameters. An advantage of our hierarchical formulation is that conjugacy can be exploited to integrate ***ϕ*** out, obtaining the Dirichlet–Multinomial model, ***y***_*i*_∼DM(***γ***), with probability mass function

$$\begin{array}{*{20}l} f\left(\mathbf{y} \mid \boldsymbol{\gamma}\right) &= \frac{\Gamma\left(y_{+} + 1\right)\Gamma\left(\gamma_{+}\right)}{\Gamma\left(y_{+} + \gamma_{+}\right)}\\ & \quad\times \prod_{j = 1}^{J}\frac{\Gamma\left(y_{j} + \gamma_{+}\right)}{\Gamma(\gamma_{j})\Gamma\left(y_{j} + 1\right)}. \end{array} $$

where $\gamma _{+} = \sum _{j}^{J} \gamma _{j}$. First described in [23] as the compound multinomial, the DM(***γ***) allows more flexibility than the Multinomial when encountering overdispersion in multivariate count data, as it induces an increase in the variance by a factor of (*y*_+_+*γ*_+_)/(1+*γ*_+_).

Next, we incorporate the covariates into the modeling via a log-linear regression framework where the DM parameters depend on the available covariates **X**’s. More specifically, we define *ζ*_*j*_= log(*γ*_*j*_) and assume

2 $$\begin{array}{*{20}l} \zeta_{j} = \alpha_{j} + \sum_{p = 1}^{P} \beta_{pj} \, \mathbf{x}_{p},  \end{array} $$

*i*=1,…,*n*; *j*=1,…,*J*. In this formulation, the intercept term *α*_*j*_ corresponds to the log baseline parameter for the taxon *j*, whereas the regression parameter *β*_*pj*_ captures the effect of the *p*th covariate on the abundance for that taxon.

Identifying the significant associations between taxa and covariates in model (1)–(2) is equivalent to determining the non-zero *β*_*pj*_ parameters. One way to address this issue is through variable selection and the use of *spike-and-slab* mixture priors [32, 33]. First, we introduce latent binary indicator vectors ***ξ***_*j*_=(*ξ*_1*j*_,*ξ*_2*j*_,…,*ξ*_*pj*_), such that *ξ*_*pj*_=1 if the *p*th covariate influences the abundance of the *j*th taxa and *ξ*_*pj*_=0 otherwise. Then, we write the prior on the *β*_*pj*_’s as

3 $$\begin{array}{*{20}l} \beta_{pj} \sim \xi_{pj} \, \mathcal{N}\left(0, r^{2}_{j}\right) + \left(1 - \xi_{pj}\right)\, \delta_{0}\left(\beta_{pj}\right), \end{array} $$

where *δ*_0_ denotes a Dirac-delta at 0 and $r^{2}_{j}$ is some suitably large value [34, 35]. It is common to choose relatively large values for $r^{2}_{j}$. Such a choice suggest a flat prior distribution on the location of the coefficients {*β*_*pj*_∣*ξ*_*pj*_=1} and therefore encourages the selection of relatively large effects. In the Results Section, we discuss the results of a sensitivity analysis to assist with the choice of this parameter. We place Bernoulli priors on the selection indicators *ξ*_*pj*_, that is

4 $$\begin{array}{*{20}l} \pi\left(\boldsymbol{\xi}_{j} \mid \mathbf{p}_{j}\right) = \prod_{p = 1}^{P} p_{pj}^{\xi_{pj}}\left(1 - p_{pj}\right)^{1 - \xi_{pj}}. \end{array} $$

**Fig. 1:**
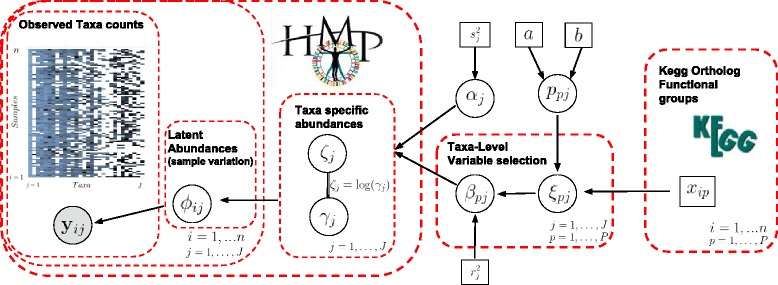
Schematic overview of the proposed integrative Bayesian approach for the application to data from the Human Microbiome Project. The observed data counts (*right*) are regressed on the available covariates (*left*), through a variable selection approach, which informs the (unknown) population abundance of each taxon

We also specify Beta hyperpriors on the hyperparameters *p*_*pj*_, i.e., *p*_*pj*_∼Beta(*ab*), as this has been shown to provide an automatic adjustment for multiplicity [36]. This is equivalent to placing a Beta mixed Binomial distribution on *ξ*_*pj*_,

$$\pi\left(\xi_{pj}\right) = \int \pi\left(\xi_{pj} \mid \lambda\right) \pi(\lambda) d\lambda, $$

with *λ*=(*a,b*). As a practical suggestion, the hyper-parameters *a* and *b* should be chosen so to induce a relatively weakly specification of the prior as a “flat” Beta distribution. This can be obtained by setting *a* and *b* so that *a*+*b*=2, and the prior expected mean value *m*=*a*/(*a*+*b*). For most cases, a value of *m*=0.01, which corresponds to assuming a priori that 1% of the *P* covariates will be selected, provides an adequate balance between false positives and false negative counts, as we further illustrate in a sensitivity analysis in the Results Section. Finally, we assume normal priors on the *α*_*j*_’s, i.e. $\alpha _{j}\sim \mathcal {N}(0, s^{2}_{j})$. Large values for $s^{2}_{j}$ encode a diffuse prior, to describe non-informative or objective prior beliefs. However, results are typically quite robust to prior choices on the intercept parameters, and $s^{2}_{j}=10$ is usually assumed as a default specification in Bayesian regression when dealing with standardized variables. Figure 1 provides an overview of the proposed integrative modeling approach, with reference to the application to the Human Microbiome Project data we describe later.

### MCMC algorithm

We implement a stochastic search Markov Chain Monte Carlo (MCMC) algorithm for posterior inference that employs a Gibbs scan to sample the non-zero regression coefficients [37]. We encourage an efficient sampling by employing a component-wise adaptive Metropolis algorithm [38] as described below. A generic iteration of the MCMC algorithm comprises the following steps:

1. **Update of** ***α*** **:** This is a Metropolis-Hastings step with a symmetric random walk proposal $\alpha _{j}^{'} \sim \mathcal {N}(\alpha _{j}, t_{\alpha }^{2})$, for *j*=1,…,*J*.
2. **Joint update of (** ***ξ*** **,** ***β*** **):** We sample these parameters jointly via a Gibbs scan that employs a Metropolis acceptance step. For each *j*=1,…,*J* and *p*=1,…,*P*:
*if* *ξ*_*pj*_=1: propose $\xi _{pj}^{'} = 0$ and $\beta _{pj}^{'} = 0$.
*if* *ξ*_*pj*_=0: propose $\xi _{pj}^{'} = 1$ and then propose $\beta _{pj}^{'}$ following an adaptive Metropolis-Hasting scheme
    $$\begin{array}{*{20}l} \beta_{pj}^{'} \sim&\, 0.95 \, \mathcal{N}\left(\beta_{pj}, 2.38^{2} \times \hat{\sigma}^{2}_{\beta_{pj}} / J\times P\right)\\ &+ 0.05 \, \mathcal{N}\left(\beta_{pj}, 0.01 / J\times P\right), \end{array} $$
    where $\hat {\sigma }^{2}_{\beta _{pj}}$ is the current estimate of the variance of the target distribution. The value of $\hat {\sigma }^{2}_{\beta _{pj}}$ is updated using a recursive formula as in [39] on all the previous draws for *β*_*pj*_.
  Accept ($\xi _{pj}^{'}, \beta _{pj}^{'}$) with probability
    $$a = \min \left\{ 1, \frac{\pi\left(\xi_{pj}^{'}, \beta_{pj}^{'} \mid \boldsymbol{\xi}_{pj}^{'}, \boldsymbol{\beta}_{pj}^{'}, \text{else}\right) }{ \pi\left(\xi_{pj}, \beta_{pj} \mid \boldsymbol{\xi}_{pj}^{'}, \boldsymbol{\beta}_{pj}^{'}, \text{else}\right)} \right\}, $$
    where $\boldsymbol {\xi }_{pj}^{'} = (\xi _{1,j}^{'}, \dots, \xi _{p-1,j}^{'}, \xi _{p + 1,j}, \dots, \xi _{pj})$, and $\boldsymbol {\beta }_{pj}^{'} = (\beta _{1,j}^{'}, \dots, \beta _{p-1,j}^{'}, \beta _{p + 1,j}, \dots, \beta _{pj})$.

For posterior inference, we are interested in identifying the relevant associations between taxa and covariates as captured by the selection indicators *ξ*_*pj*_’s and the corresponding regression coefficients *β*_*pj*_’s. Estimates of the marginal posterior probabilities of inclusion (PPIs) of the latent indicators *ξ*_*pj*_ can be calculated by counting the number of times that each taxa/covariate association is included across the MCMC iterations. A selection of the significant associations can then be made by choosing those elements that have marginal PPIs greater than a specific value, for example greater than 0.5 for the median probability model of [40]. Another choice for the threshold which controls for multiplicity [41] relies on an estimated pre-specified Bayesian false discovery rate *α* calculated as

$$\widehat{\text{FDR}}(c) = \frac{\sum_{p = 1}^{P} \sum_{j = 1}^{J} \left(1 - \widehat{\text{PPI}}_{pj}\right) D_{pj}}{\sum_{p = 1}^{P} \sum_{j = 1}^{J} D_{pj}}, $$

where . An optimal threshold *c* ^′^ can be found for error rate *α* by choosing *c* ^′^ such that $\widehat {\text {FDR}}(c') < \alpha $. Estimates of the non-zero regression coefficients *β*_*pj*_ can also be calculated by averaging over the sampled MCMC values.

In order to compare selection performance of different methods, we calculate accuracy, false positive rate (FPR), false negative rate (FNR) and Matthews correlation coefficient (MCC), across 30 replicated datasets. We define accuracy as ACC = (TP + TN)/(P + N), with TP the number of true positives out of P selected and TN the number of true negatives out of N not selected. The false negative rate is calculated as FNR = FN/(FN + TP), the false positive rate as FPR = FP/(FP + TN), and the Matthews correlation coefficient as

$$\begin{array}{*{20}l} \text{MCC} =\text{MCC} = \frac{TP / N - S \times P} {\sqrt{PS (1 - S) (1 - P)} }, \end{array} $$

with N=*TN*+*TP*+*FN*+*FP*, $\mathrm {P} = \frac {TP + FP} { N }$ and $\mathrm {S} = \frac {TP + FN} { N }$ [42]. Since the MCC balances TP and FP counts, and can be used even if the classes are of very different sizes, it is generally regarded as one of the most appropriate measures of classification accuracy. We further computed receiving operating curves (ROC) to compare the performance of the selection procedure across the different methods.

### Comparison study on simulated data

We carry out a simulation study to assess the performance of our model and compare results to alternative methods. More specifically, we consider two methods which have been specifically employed for the integrative analysis of microbiome data: the penalized approach of Chen and Li [26], and the false discovery rate-corrected pair-wise correlation tests considered in [19]. In addition, we consider the factorized maximum a posteriori (MAP) Bayesian lasso of [43], a recently proposed general statistical method for conducting variable selection in multivariate count-response regression. When fitting the Bayesian Gamma Lasso method of [43], model selection was done using the minimum AIC, while for Chen and Li’s approach the minimum BIC was calculated with the group penalty set to 20%. We also fit the method of Chen and Li to the untransformed data. The false discovery rate threshold for the Spearman’s correlation tests was set to 0.05.

In simulating data, we set *n*=100, *P*=50 and *J*=50, and chose *P*_*r*_=9 and *J*_*r*_=5 to obtain a total number of relevant taxa/covariate associations equal to 25. We simulated the covariate matrix **X** according to a Multivariate-Normal (0,*Σ*) with *Σ*_*i,j*_=*ρ* ^|*i*−*j*|^ and *ρ*=0.4. We drew each vector **y**_*i*_ of counts from a Dirichlet-Multinomial distribution as follows. For *i*=1,…,*n*, $\mathbf {y}_{i}\sim \text {Multinomial}(N_{i},\boldsymbol {\pi }_{i}^{*})$, with the row sum *N*_*i*_∼*DiscreteUnif*[1,0000;2,000], and $\boldsymbol {\pi }^{*}_{i}=(\pi ^{*}_{i1}, \ldots, \pi ^{*}_{iJ})\sim \text {Dirichlet}(\boldsymbol {\gamma }^{*})$. For $\boldsymbol {\gamma }^{*}=(\gamma ^{*}_{1},\dots,\gamma ^{*}_{J})$, we set $\gamma _{j}^{*}=\frac {\gamma _{j}}{\gamma _{+}}\,\frac {1-\psi }{\psi }$, *j*=1,…,*J*, with *γ*_*j*_= exp{*α*_*j*_+**X** ***β***_*j*_}, $\gamma _{+} = \sum _{j = 1}^{J} \gamma _{j}$ and *ψ*∈ [ 0,1] an overdispersion parameter. When *ψ*→0, the simulated values approximate a Multinomial (***π***) distribution, while for large *ψ*, the sampled values are more disperse. Here, we set *ψ*=0.01. We sampled the non-zero *β*_*pj*_’s from the intervals ±[ 0.5,1.0] and the intercept parameters from a Uniform(–2.3, 2.3). Below we report performance results as averages over 30 replicated simulated datasets.

When running the MCMC, we used a vague prior for the intercept by setting the variance parameter to $s_{pj}^{2} = 10$. Similarly, we set $r_{pj}^{2} = 10$, to provide sufficiently vague prior information on the non-zero log-linear regression coefficients. Finally, we set *m*=0.01 (or *a*=0.02 and *b*=1.98), resulting in a sparse prior mean on selected associations of 1% of the total. We provide comments on the sensitivity of the selection results to the choice of these hyperparameters in the Section below. We ran the MCMC algorithm for 10,000 iterations and thinned to every fifth iteration. On a single dataset, the C code took approximately 31.5 min to run on an Intel Xeon E5-2630 2.30 GHz processor. We assessed convergence visually and via the Geweke diagnostic [44] as implemented in the R package coda. Convergence was checked for a) the number of active variables in each iteration and b) the samples from each of the selected *β*_*pj*_. The five number summary of the 25 Geweke *z*-scores was (–3.43, –1.06, –0.63, 0.71, 1.98).

### Inferring associations between taxonomic abundances and metabolic pathways

We demonstrate our approach on publicly available data obtained from the Human Microbiome Project (HMP) website [29] from which we use 79 samples from healthy individuals. The **Y** matrix in our model contains 16S rRNA microbial counts from stool samples at the genus taxonomic level. As common in microbiome studies, the genera abundances (*Bacteroides*, *Prevotella*, etc.) were filtered by requiring each genus to be present in *at least* 5% of the samples. This procedure removes extremely low-abundance genera leaving 80 genera for the analysis. From the same 79 individuals, we obtained KEGG orthology group abundances which are used as the matrix of covariates **X** of our model. The KEGG orthology groups were reconstructed from metagenomic shotgun sequencing (WGS) using the HMP Unified Metabolic Analysis Network (HUMAnN) pipeline [45] and were also provided on the HMP website. These values represent inferred abundances of biochemical functional groups and metabolic pathways present due to the shotgun sequenced reads of bacterial and non-bacterial genes in the sample. To reduce correlation among the covariates we used average linkage clustering on the correlation matrix of the KEGG groups and chose one representative from each cluster, according to its relevance to microbiome research, leaving 76 columns in **X**. Finally, the columns in **X** were mean centered and scaled to unit variance. Though the HMP sampled 300 individuals for several timepoints and over many sites, there were relatively few samples that included the WGS used to obtain the KEGG orthology data. Thus, when joining the samples from the 16S rRNA data and the KEGG orthology data, a total of 79 matched samples remained.

We used the same hyperparameter settings as in the simulation study, that is $s_{pj}^{2} = 10$ and $r_{pj}^{2} = 10$ and set *m*=0.01, resulting in a sparse mean selection prior of 1% of the total 6,080 possible associations. The MCMC algorithm described in “MCMC algorithm” section above was run for 500,000 iterations and thinned to every 100th draw. We assessed convergence visually and via the Geweke diagnostic [44] as implemented in the R package coda. The five number summary of the Geweke *z*-scores for the 26 *β*_*pj*_’s was (–3.83, –1.19, 0.15, 1.46, 3.38).

## Results

### Simulation study

**Fig. 2:**
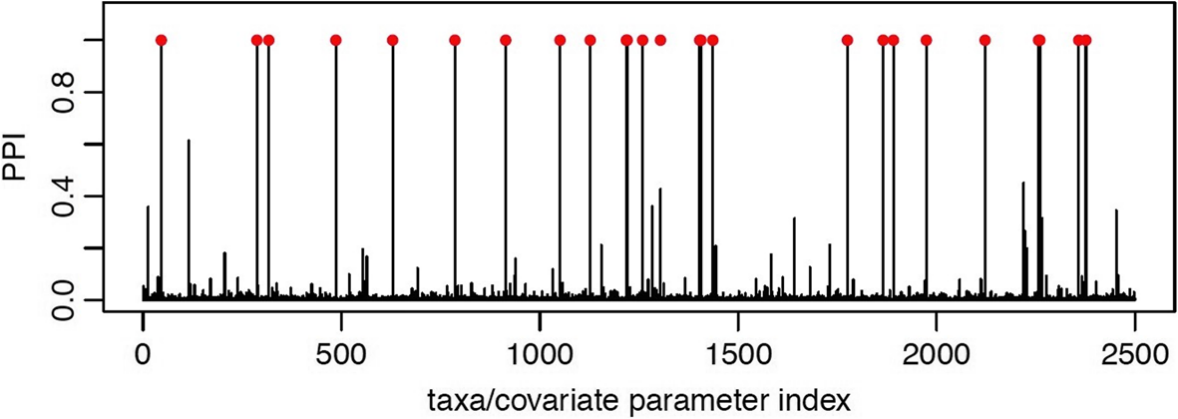
Simulated data: Marginal posterior probabilities of inclusion (PPI) for each coefficient *β* *jp*, *j*=1,…,50, *p*=1,…,50, describing the association between each taxa and each covariate. Each PPI is obtained by averaging the number of times that each taxa/covariate association is included across the MCMC iterations, after burn-in. The true associations are indicated as *red dots*

In Fig. 2 we show the plot of the marginal PPIs of the *P*×*J* elements *ξ*_*pj*_, obtained by computing the proportion of times that *ξ*_*pj*_=1 across all iterations, after burn-in. The selected median model, corresponding to a threshold of 0.5 on the PPIs, results in a false positive rate of 0.0004 and a false negative rate of 0.04. The value of the AUC for this replicate was 0.99.

**Fig. 3:**
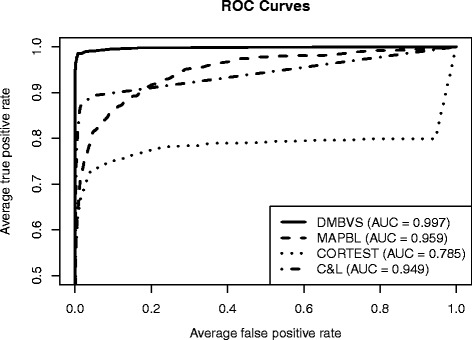
Simulated data: Comparison results of selection performances (ROC curves). DMBVS: Dirichlet–Multinomial Bayesian Variable Selection (our method), MAPBL : Maximum A Posteriori Bayesian Lasso, CORTEST: Multiplicity Corrected Correlation Tests as in Wu et al. (2010), C&L: composite penalty from Chen and Li (2013)

Figure 3 illustrates the selection performance of the proposed method, by plotting the average ROC curves over the 30 replicated datasets (*ψ*=0.01) for each of the methods included in the comparison. The Figure shows that our proposed model outperforms the competing methods in terms of achieved average true and false positive rates.

As an additional comparison, together with the total number of correctly identified regression parameters, which we term “overall recovery”, we also looked at the “taxa-wise recovery”, which we defined as the correct recovery of any element from one of the *J* taxa. Thus, recovery for overall selection occurs for *P*×*J* elements while taxa-wise selection occurs for *J* elements. Table 1 reports average values for accuracy, FPR, FNR and MCC, averaged across the 30 replicated datasets, for both overall and taxa-wise recovery. These results show that our method in particular outperforms competing methods for taxa-wise recovery. In the same Table we report results for a more challenging simulated scenario, obtained with a higher value of the overdispersion parameter (*ψ*=0.1). As expected, the increase in overdispersion makes the selection task more difficult for all methods. However, our method still outperforms or is commensurate with the competing methods, even in the presence of considerable overdispersion.

**Table 1:** Simulated data: performance assessment for two different scenarios, characterized by different values of the dispersion parameter *ψ*

	*Overall*				*Taxa*			
	DMBVS	MAPBL	C&L	CORTEST	DMBVS	MAPBL	C&L	CORTEST
*ψ*=0.01								
MCC	0.93	0.64	0.67	0.73	0.89	0.66	0.50	0.85
FNR	0.05	0.10	0.12	0.31	0.00	0.46	0.43	0.02
FPR	0.00	0.01	0.01	0.00	0.05	0.01	0.09	0.06
Accuracy	1.00	0.99	0.99	1.00	0.96	0.91	0.85	0.95
*ψ*=0.1								
MCC	0.72	0.42	0.54	0.56	0.73	0.40	0.38	0.70
FNR	0.39	0.58	0.28	0.63	0.24	0.73	0.52	0.37
FPR	0.00	0.01	0.01	0.00	0.05	0.01	0.12	0.02
Accuracy	1.00	0.99	0.99	1.00	0.92	0.86	0.81	0.92

Values are rounded averages over thirty replicates. Results for Matthews’ Correlation Coefficient, Falso Positive Rate, False Negative Rate, and Accuracy, are based on the median probability model. DMBVS: Dirichlet–Multinomial Bayesian Variable Selection (our method), MAPBL: Maximum A Posteriori Bayesian Lasso, C&L: composite penalty from Chen and Li (2013), CORTEST: Multiplicity Corrected Correlation Tests as in Wu et al. (2010)

#### Sensitivity analysis

Since our proposal requires the choice of a number of hyperparameters, it is important to investigate how sensitive the results are to varying parameter sets. Therefore, we conclude our simulation study by briefly discussing the sensitivity of the results to the prior specifications. In general, we found that results were robust to the prior choices on the intercept parameters, *α*_*j*_, while, as expected, some sensitivity was observed with respect to the variance hyperparameters of the *spike-and-slab* prior (3) on the regression coefficients, *β*_*pj*_, and the hyperparameters of the Beta priors on *p*_*pj*_. In Table 2 we report results obtained by considering a full grid of values for the prior expected value of *p*_*pj*_, i.e. *m*∈{0.005,0.01,0.05}, and the slab variance, $r^{2}_{pj} \in \{1, 10, 100 \}$. In the Additional file 1, we further report the corresponding ROC curves. With only 25 truly non-zero *β*_*pj*_’s, out of 2,500 parameters, small increases in false positive rates can drastically decrease the Matthews correlation coefficient. Thus imposing some sparsity by using a smaller value for *m* improves overall performance while larger values of *m* allow for more false positives. The results appear to suggest that assuming moderate sparsity a priori (e.g., *m*=0.01) generally leads to good operating characteristics. Similarly, when the slab variance is small, e.g. $r_{pj}^{2} = 1$, there is more prior density close to zero, which allows small but insignificant variables to be selected. Conversely, when the slab variance is large, e.g. $r_{pj}^{2} = 100$, false positives are less likely but false negatives increase, since the prior density is spread more evenly over the support. Therefore, an intermediate value, e.g. $r_{pj}^{2} = 10$, provides a reasonable compromise, which favors relatively large effect sizes and a small number of false positives. In the Additional file 1, we also report the performance of our method for varying values of the over-dispersion parameter *ψ* and the sample size *n*. As expected, the results show that the performances improve for larger sample sizes and decreasing overdispersion.

**Table 2:** Simulated data: sensitivity analysis for varying values of the prior expected value of *p*_*pj*_, *m*, and the slab variance $r^{2}_{pj}$, and for two different scenarios, characterized by different values of the dispersion parameter *ψ*

	*m*=0.005			*m*=0.01			*m*=0.05		
	$r_{pj}^{2} = 1$	$r_{pj}^{2} = 10$	$r_{pj}^{2} = 100$	$r_{pj}^{2} = 1$	$r_{pj}^{2} = 10$	$r_{pj}^{2} = 100$	$r_{pj}^{2} = 1$	$r_{pj}^{2} = 10$	$r_{pj}^{2} = 100$
*ψ*=0.01									
MCC	0.69	0.93	0.96	0.69	0.93	0.95	0.69	0.93	0.95
FPR	0.01	0.00	0.00	0.01	0.00	0.00	0.01	0.00	0.00
FNR	0.02	0.04	0.08	0.02	0.05	0.09	0.02	0.05	0.08
Accuracy	0.99	1.00	1.00	0.99	1.00	1.00	0.99	1.00	1.00
AUC	1.00	1.00	1.00	1.00	1.00	1.00	1.00	1.00	1.00
*ψ*=0.1									
MCC	0.53	0.73	0.72	0.53	0.73	0.71	0.52	0.73	0.72
FPR	0.01	0.00	0.00	0.01	0.00	0.00	0.01	0.00	0.00
FNR	0.26	0.37	0.46	0.26	0.37	0.47	0.26	0.37	0.47
Accuracy	0.99	1.00	1.00	0.99	1.00	1.00	0.99	1.00	1.00
AUC	0.96	0.96	0.93	0.96	0.96	0.93	0.95	0.95	0.93

Values are averages over 30 replicates

### Data analysis

**Fig. 4:**
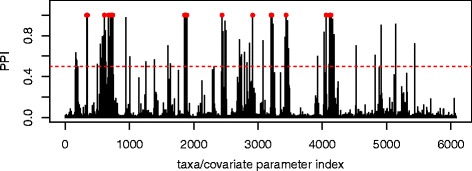
Real data: Marginal posterior probabilities of inclusion (PPI) for each coefficient *β* *jp*, in Eq. (2), describing the association between each taxa and each covariate. Each PPI is obtained by averaging the number of times that each taxa/covariate association is included across the MCMC iterations, after burn-in. Here, the median model, corresponding to a threshold of 0.5 on the PPIs, selects 92 associations. Among those, 26 have a marginal PPI greater than 0.98, which corresponds to a Bayesian FDR of 0.1. These 26 associations are indicated as *red dots*

**Fig. 5:**
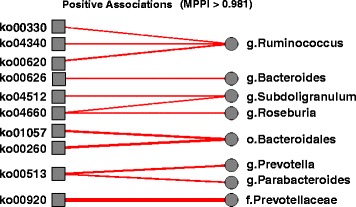
Real data: Selected positive taxa-by-covariate associations. The magnitude of the association, as captured by the median of the MCMC draws for each *β*_*pj*_, is proportional to the width of the edges

**Fig. 6:**
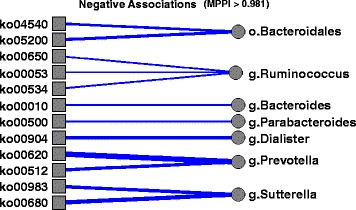
Real data: Selected negative taxa-by-covariate associations. The magnitude of the association, as captured by the median of the MCMC draws for each *β*_*pj*_, is proportional to the width of the edges

Figure 4 shows the traceplot of the number of included taxa/covariate associations and the plot of the marginal PPIs of the *P*×*J* elements *ξ*_*pj*_, obtained by computing the proportion of times that *ξ*_*pj*_=1 across all iterations, after burn-in. Here the median model, corresponding to a threshold of 0.5 on the PPIs, selects 92 associations. Among those, 26 have a marginal PPI greater than 0.98, which corresponds to a Bayesian FDR of 0.1. These 26 associations are listed in Table 3, together with the corresponding estimated regression coefficients, and depicted in Figs. 5 and 6, for positive and negative associations, respectively. In these Figures, the magnitude of the association, as captured by the estimated *β*_*pj*_’s, is proportional to the width of the edges, with red lines indicating negative associations and blue lines positive associations. As a comparison, the method by Chen and Li [26] identified 120 associations, whereas the Bayesian Lasso of [43] and the correlation test-based method of [19] identified, respectively, 220 and 711 associations. Those results appear to confirm the sparser selection achieved by our method, consistently with the results of the simulation study.

**Table 3:** Real data: selection results using a BFDR of 0.1

KEGG ID	Pathway	Taxa	MPPI	*β*_*pj*_
ko04660	T cell receptor signaling pathway	g.Subdoligranulum	1.00	0.40
ko04512	ECM-receptor interaction	g.Subdoligranulum	1.00	0.44
ko00680	Methane metabolism	g.Sutterella	1.00	-1.47
ko05200	Pathways in cancer	o.Bacteroidales	1.00	-1.04
ko04540	Gap junction	o.Bacteroidales	1.00	-0.92
ko00534	Glycosaminoglycan biosynthesis	g.Ruminococcus	1.00	-0.56
ko00053	Ascorbate and aldarate metabolism	g.Ruminococcus	1.00	-0.46
ko00650	Butanoate metabolism	g.Ruminococcus	1.00	-0.55
ko00513	Various types of N-glycan biosynthesis	g.Parabacteroides	1.00	0.54
ko00500	Starch and sucrose metabolism	g.Parabacteroides	1.00	-0.61
ko00904	Diterpenoid biosynthesis	g.Dialister	1.00	-1.21
ko00360	Phenylalanine metabolism	g.Dialister	1.00	-2.18
ko00626	Naphthalene degradation	g.Bacteroides	1.00	0.39
ko00010	Glycolysis / Gluconeogenesis	g.Bacteroides	1.00	-0.57
ko00513	Various types of N-glycan biosynthesis	g.Prevotella	1.00	0.77
ko00512	Mucin type O-Glycan biosynthesis	g.Prevotella	1.00	-1.06
ko00620	Pyruvate metabolism	g.Prevotella	1.00	-1.76
ko04340	Hedgehog signaling pathway	g.Ruminococcus	1.00	0.45
ko04660	T cell receptor signaling pathway	g.Roseburia	1.00	0.46
ko00983	Drug metabolism	g.Sutterella	1.00	-1.20
ko00260	Glycine, serine and threonine metabolism	o.Bacteroidales	1.00	0.88
ko00330	Arginine and proline metabolism	g.Ruminococcus	0.99	0.39
ko01057	Biosynthesis of type II polyketide products	o.Bacteroidales	0.99	0.76
ko00620	Pyruvate metabolism	g.Ruminococcus	0.99	0.56
ko00920	Sulfur metabolism	f.Prevotellaceae	0.99	1.36
ko00010	Glycolysis/Gluconeogenesis	o.Clostridiales	0.98	0.54

The text in the KEGG column is hyperlinked to the KEGG orthology database for a more complete description of the selected pathways. Taxa names start with “g.”, “f.” or “o.” which stand for genus, family, or order, respectively, and correspond to the lowest taxonomic classification available

## Discussion

A close investigation of the biological significance of the associations identified by our model reveals several interesting characteristics and affirms the relevance of these associations. Commensal microbiota that inhabit the human gut are proficient at scavenging glycans and polysaccharides, including those in plants, such as starches or cellulose, animal-derived tissues (glycosaminoglycans and N-linked glycans), and glycans from host mucus (O-linked glycans) [46]. *Ruminococcus* spp. are known to participate in both resistant starch and glycosaminoglycan degradation [46, 47]. It has been reported that long-term consumption of diets rich in protein and animal fat were associated with an enterotype primarily containing increased *Bacteroides* and *Ruminococcus* species [19]. Additionally, *Ruminococcus torques* and *Ruminococcus gnavus* have been shown to degrade mucins [48]. Thus, it is logical that *Ruminococcus*, which is one of the noteworthy genera involved in glycosaminoglycan degradation, would be negatively associated to glycosaminoglycan biosynthesis (ko00534) (Table 3). Similarly, *Parabacteroides* which is also negatively associated with N-glycan biosynthesis (ko00513), is involved in deglycosylation and utilization of N-glycans [49]. Also, among the associations identified for the glycan pathways, *Prevotella* was negatively associated with mucin type O-glycan biosynthesis (ko00512). In the literature, *Prevotella* has implications for mucosal homeostasis, as some *Prevotella* spp. express a unique mucin-desulfating glycosidase that can hydrolyze GlcNAc residues on mucin-type O-glycans, and thus is important for mucin degradation [50]. Other associations affirmed through the literature included that of *Bacteroides* with naphthalene degradation (ko00626). It has been reported that *Bacteroidetes* possess the capability to degrade polycyclic aromatic hydrocarbons such as naphthalene [51]. Associations of *Ruminococcus* with pyruvate metabolism (ko00620) are also supported, as phosphoenolpyruvate carboxykinase was previously reported to be associated with *Ruminococcus flavefaciens* in the rumen [52]. Another supported association is that of *Prevotellaceae* with sulfur metabolism. L-cysteine desulfhydrase enzymes have been characterized in *Prevotella intermedia* [53]. Additionally, glycosulfatase enzymes have been described in *Prevotella* [54]. Equally interesting is the selection of pathways that are expected to be omnipresent among many bacteria, such as glycolysis/gluconeogenesis (ko00010), as glycolysis occurs, with variations, in nearly all organisms, both aerobic and anaerobic. Thus, it is not surprising that taxa like *Clostridiales* are positively associated with glycolysis/gluconeogenesis as they are abundant taxa within the gut microbiome.

Given the complexity of metabolic pathways and the process of mapping specific genes to pathways, some of the selected associations are unexpected, and might be due to the 16S abundances that were made available at the HMP site and the mapping of metagenomic sequences to specific KEGG orthology groups by HUMAnN. For example, several species of *Ruminococcus* are known to participate in butanoate (butyrate) metabolism [55], *Dialister* spp. have phenylalanine arylamidase activities [56], and *Prevotella* spp. are known to participate in pyruvate metabolism [57, 58]. Since those associations should be driven exclusively by bacterial genes, it is interesting that we find significant associations between the abundance of certain bacterial taxa and KEGG pathways that are primarily reported among eukaryotic species (i.e., T-cell receptor signaling, hedgehog signaling, pathways in cancer, etc.). Indeed, although precautionary steps are performed, the HMP consortium reported that human contaminants are found in 50–90% of the sequences [15]. This might also explain the negative association exhibited by *Bacteriodes* and glycolysis/gluconeogenesis. These unexpected findings suggest the need for further investigations and validation.

## Conclusion

Herein, we have developed a Bayesian approach to the Dirichlet-Multinomial regression models that allows for the selection of significant associations between covariates and taxa from a microbiome abundance table by imposing *spike-and-slab* priors on the log-linear regression coefficients of the model. We have applied our model to simulated data and compared performances with methods developed for similar applications. We have illustrated the performance of our method using publicly available data on taxonomic abundances and metabolic pathways inferred from whole genome shotgun sequencing reads, which we obtained from the Human Microbiome Project website. Our results have revealed interesting links between specific taxa (i.e. genera) and particular metabolic pathways, which we have validated via existing literature.

Several extensions of our model are possible. Because some habitats, e.g. the gut, are thought to have highly variable dynamics, longitudinal sampling may be preferred to cross-sectional sampling since it may give a better sense of long-term trends [59]. Thus, incorporating repeated samples with specified correlation structures in the linear predictor could produce additional insights. Another important aspect of microbiome data, which is receiving attention from researchers, is the heterogeneity in community structure across samples, as this can be an indication of the existence of “enterotypes” [60, 61]. This can be addressed within our modeling framework by employing Bayesian nonparametric models that would allow to cluster selected associations across partitions of the samples. These extensions are currently under investigation.

## Additional files

Additional file 1 Comparison with other methods and sensitivity analysis in the simulation study. (PDF 142 kb)

Additional file 2 The dataset comprising the 16S rRNA microbial counts. (ZIP 1280 kb)

Additional file 3 The dataset used for the KEGG orthology groups. (ZIP 718 kb)

## Abbreviations

AIC: Akaike information criterion
ACC: True positive
AUC: Area under the curve
BIC: Bayesian information criterion
DM: Dirichlet-Multinomial
FDR: False discovery rate
FN: False negative
FNR: False negative rate
FP: False positive
FPR: False positive rate
N: Negative
HMP: Human Microbiome Project
KEGG: Kyoto encyclopedia of genes and genomes
MAP: Maximum a posteriori
MCC: Matthews correlation coefficient
MCMC: Markov Chain Monte Carlo
OTU: Operational taxonomic unit
P: Positive
PPI: Posterior probability of inclusion
ROC: Receiving operating curve
TN: True negative
WGS: Whole genome shotgun sequencing

## References

[1] Morgan XC, Huttenhower C (2012). Chapter 12: Human microbiome analysis. PLoS Comput Biol.

[2] Zhu B, Wang X, Li L (2010). Human gut microbiome: The second genome of human body. Protein Cell.

[3] Grice EA, Segre JA (2012). The Human Microbiome: our second genome. Annu Rev Genomics Hum Genet.

[4] Fraher MH, O’Toole PW, Quigley EMM (2012). Techniques used to characterize the gut microbiota: a guide for the clinician. Nat Rev Gastroenterol Hepatol.

[5] Abraham C, Cho JH (2009). Inflammatory bowel disease. N Engl J Med.

[6] Qin J, Li Y, Cai Z, Li S, Zhu J, Zhang F, Liang S, Zhang W, Guan Y, Shen D, Peng Y, Zhang D, Jie Z, Wu W, Qin Y, Xue W, Li J, Han L, Lu D, Wu P, Dai Y, Sun X, Li Z, Tang A, Zhong S, Li X, Chen W, Xu R, Wang M, Feng Q, Gong M, Yu J, Zhang Y, Zhang M, Hansen T, Sanchez G, Raes J, Falony G, Okuda S, Almeida M, LeChatelier E, Renault P, Pons N, Batto JM, Zhang Z, Chen H, Yang R, Zheng W, Li S, Yang H, Wang J, Ehrlich SD, Nielsen R, Pedersen O, Kristiansen K, Wang J (2012). A metagenome-wide association study of gut microbiota in type 2 diabetes. Nature.

[7] Koeth RA, Wang Z, Levison BS, Buffa JA, Org E, Sheehy BT, Britt EB, Fu X, Wu Y, Li L, Smith JD, DiDonato JA, Chen J, Li H, Wu GD, Lewis JD, Warrier M, Brown JM, Krauss RM, Tang WHW, Bushman FD, Lusis AJ, Hazen SL (2013). Intestinal microbiota metabolism of L-carnitine, a nutrient in red meat, promotes atherosclerosis. Nat Med.

[8] Cryan JF, O’Mahony SM (2011). The microbiome-gut-brain axis: from bowel to behavior. Neurogastroenterol Motil.

[9] Kong HH, Oh J, Deming C, Conlan S, Grice EA, Beatson MA, Nomicos E, Polley EC, Komarow HD, Program NCS, Murray PR, Turner ML, Segre JA (2012). Temporal shifts in the skin microbiome associated with disease flares and treatment in children with atopic dermatitis. Genome Res.

[10] Romero R, Hassan SS, Gajer P, Tarca AL, Fadrosh DW, Bieda J, Chaemsaithong P, Miranda J, Chaiworapongsa T, Ravel J (2014). The vaginal microbiota of pregnant women who subsequently have spontaneous preterm labor and delivery and those with a normal delivery at term. Microbiome.

[11] Devaraj S, Hemarajata P, Versalovic J (2013). The human gut Microbiome and body metabolism: implications for obesity and diabetes. Clin Chem.

[12] Ash C, Mueller K (2016). Manipulating the Microbiota. Science.

[13] Tyler AD, Smith MI, Silverberg MS (2014). Analyzing the human Microbiome: A “How To” guide for physicians. Am J Gastroenterol.

[14] Lange A, Jost S, Heider D, Bock C, Budeus B, Schilling E, Strittmatter A, Boenigk J, Hoffmann D (2015). Ampliconduo: A split-sample filtering protocol for high-throughput amplicon sequencing of microbial communities. PLoS ONE.

[15] The Human Microbiome Project (2012). A framework for human microbiome research. Nature.

[16] McMurdie PJ, Holmes S (2014). Waste not, want not: why rarefying microbiome data is inadmissible. PLoS Comput Biol.

[17] Grossmann L, Jensen M, Heider D, Jost S, Glucksman E, Hartikainen H, Mahamdallie SS, Gardner M, Hoffmann D, Bass D, Boenigk J (2016). Protistan community analysis: key findings of a large-scale molecular sampling. ISME J.

[18] Caporaso JG, Kuczynski J, Stombaugh J, Bittinger K, Bushman FD, Costello EK, Fierer N, Peña AG, Goodrich JK, Gordon JI, Huttley GA, Kelley ST, Knights D, Koenig JE, Ley RE, Lozupone CA, Mcdonald D, Muegge BD, Pirrung M, Reeder J, Sevinsky JR, Turnbaugh PJ, Walters WA, Widmann J, Yatsunenko T, Zaneveld J, Knight R (2010). QIIME allows analysis of high-throughput community sequencing. Nature.

[19] Wu GD, Chen J, Hoffmann C, Bittinger K, Chen YY, Keilbaugh SA, Bewtra M, Knights D, Walters WA, Knight R, Sinha R, Gilroy E, Gupta K, Baldassano R, Nessel L, Li H, Bushman FD, Lewis JD (2011). Linking long-term dietary patterns with gut microbial enterotypes. Science.

[20] Youmans BP, Ajami NJ, Jiang Z-d, Campbell F, Wadsworth WD, Petrosino JF, Dupont HL, Highlander SK (2015). Characterization of the human gut microbiome during travelers’ diarrhea. Gut Microbes.

[21] Hamady M, Lozupone CA, Knight R (2010). Fast UniFrac: facilitating high-throughput phylogenetic analyses of microbial communities including analysis of pyrosequencing and PhyloChip data. ISME J.

[22] Fukuyama J, McMurdie PJ, Dethlefsen L, Relman DA, Holmes S (2017). Comparisons of distance methods for combining covariates and abundances in microbiome studies. Pac Symp Biocomput.

[23] Mosimann JE (1962). On the compound multinomial distribution, the multivariate *β*-distribution, and correlations among proportions. Biometrika.

[24] la Rosa PS, Brooks JP, Deych E, Boone EL, Edwards DJ, Wang Q, Sodergren E, Weinstock G, Shannon WD (2012). Hypothesis testing and power calculations for taxonomic-based human microbiome data. PLoS ONE.

[25] Holmes I, Harris K, Quince C (2012). Dirichlet multinomial mixtures: Generative Models for Microbial Metagenomics. PLoS ONE.

[26] Chen J, Li H (2013). Variable selection for sparse Dirichlet-multinomial regression with an application to microbiome data analysis. Ann Appl Stat.

[27] Chen J, Bushman FD, Lewis JD, Wu GD, Li H (2013). Structure-constrained sparse canonical correlation analysis with an application to microbiome data analysis. Biostatistics.

[28] Lin W, Shi P, Feng R, Li H (2014). Variable selection in regression with compositional covariates. Biometrika.

[29] The Human Microbiome Project (2012). Structure, function and diversity of the healthy human microbiome. Nature.

[30] Benson AK, Kelly SA, Legge R, Ma F, Low SJ, Kim J, Zhang M, Oh PL, Nehrenberg D, Hua K, Kachman SD, Moriyama EN, Walter J, Peterson DA, Pomp D (2010). Individuality in gut microbiota composition is a complex polygenic trait shaped by multiple environmental and host genetic factors. PNAS.

[31] Goodrich JK, Davenport ER, Waters JL, Clark AG, Ley RE (2016). Cross-species comparisons of host genetic associations with the microbiome. Science.

[32] George EI, McCulloch RE (1997). Approaches for Bayesian Variable Selection. Stat Sin.

[33] Brown PJ, Vannucci M, Fearn T (1998). Multivariate Bayesian variable selection and prediction. J R Stat Soc Ser B Stat Methodol.

[34] Smith M, Kohn R (1996). Nonparametric regression using Bayesian variable selection. J Econ.

[35] Chipman H, George EI, Mcculloch RE (2001). The Practical Implementation of Bayesian Model Selection. IMS Lect Notes - Monogr Ser.

[36] Scott JG, Berger JO (2010). Bayes and empirical-Bayes multiplicity adjustment in the variable-selection problem. Ann Stat.

[37] Savitsky T, Vannucci M, Sha N (2011). Variable selection for nonparametric gaussian process priors: models and computational strategies. Stat Sci.

[38] Roberts GO, Rosenthal JS (2009). Examples of Adaptive MCMC. J Comput Graph Stat.

[39] Haario H, Saksman E, Tamminen J (2005). Componentwise adaptation for high dimensional MCMC. Comput Stat.

[40] Barbieri MM, Berger JO (2004). Optimal predictive model selection. Ann Stat.

[41] Newton MA, Noueiry A, Sarkar D, Ahlquist P (2004). Detecting differential gene expression with a semiparametric hierarchical mixture method. Biostatistics.

[42] Matthews BW (1975). Comparison of the predicted and observed secondary structure of T4 phage lysozyme. Biochim Biophys Acta.

[43] Taddy MA (2013). Multinomial inverse regression for text analysis (with discussion). J Am Stat Assoc.

[44] Geweke J (2012). Evaluating the accuracy of sampling-based approaches to the calculation of posterior moments. Bayesian Stat 4.

[45] Abubucker S, Segata N, Goll J, Schubert AM, Izard J, Cantarel BL, Rodriguez-Mueller B, Zucker J, Thiagarajan M, Henrissat B, White O, Kelley ST, Methé B, Schloss PD, Gevers D, Mitreva M, Huttenhower C (2012). Metabolic reconstruction for metagenomic data and its application to the human microbiome. PLoS Comput Biol.

[46] Koropatkin NM, Cameron EA, Martens EC (2012). How glycan metabolism shapes the human gut microbiota. Nat Rev Microbiol.

[47] Walker AW, Ince J, Duncan SH, Webster LM, Holtrop G, Ze X, Brown D, Stares MD, Scott P, Bergerat A, Louis P, McIntosh F, Johnstone AM, Lobley GE, Parkhill J, Flint HJ (2011). Dominant and diet-responsive groups of bacteria within the human colonic microbiota. ISME J.

[48] Crost EH, Tailford LE, Le Gall G, Fons M, Henrissat B, Juge N. Utilisation of Mucin Glycans by the Human Gut Symbiont Ruminococcus gnavus Is Strain-Dependent. PLoS ONE. 2013;8(10). doi:10.1371/journal.pone.0076341. 10.1371/journal.pone.0076341 PMC3808388 24204617

[49] Cao Y, Rocha ER, Smith CJ (2014). Efficient utilization of complex N-linked glycans is a selective advantage for Bacteroides fragilis in extraintestinal infections. PNAS.

[50] Rho JH, Wright DP, Christie DL, Clinch K, Furneaux RH, Roberton AM (2005). A novel mechanism for desulfation of mucin: Identification and cloning of a mucin-desulfating glycosidase (sulfoglycosidase) from Prevotella strain RS2. J Bacteriol.

[51] Hilyard EJ, Jones-Meehan JM, Spargo BJ, Hill RT (2008). Enrichment, isolation, and phylogenetic identification of polycyclic aromatic hydrocarbon-degrading bacteria from Elizabeth River sediments. Appl Environ Microbiol.

[52] Schöcke L, Weimer PJ (1997). Purification and characterization of phosphoenolpyruvate carboxykinase from the anaerobic ruminal bacterium Ruminococcus flavefaciens. Arch Microbiol.

[53] Yano T, Fukamachi H, Yamamoto M, Igarashi T (2009). Characterization of L-cysteine desulfhydrase from Prevotella intermedia. Oral Microbiol Immunol.

[54] Wright DP, Rosendale DI, Roberton AM (2000). Prevotella enzymes involved in mucin oligosaccharide degradation and evidence for a small operon of genes expressed during growth on mucin. FEMS Microbiol Lett.

[55] Takahashi K, Nishida A, Fujimoto T, Fujii M, Shioya M, Imaeda H, Inatomi O, Bamba S, Andoh A, Sugimoto M (2016). Reduced abundance of butyrate-producing bacteria species in the fecal microbial community in Crohn’s disease. Digestion.

[56] Jumas-Bilak E, Jean-Pierre H, Carlier JP, Teyssier C, Bernard K, Gay B, Campos J, Morio F, Marchandin H (2005). Dialister micraerophilus sp nov and Dialister propionicifaciens sp nov., isolated from human clinical samples. Int J Syst Evol Microbiol.

[57] Takahashi N, Yamada T (2000). Pathways for amino acid metabolism by Prevotella intermedia and Prevotella nigrescens. Oral Microbiol Immunol.

[58] Ruan Y, Shen L, Zou Y, Qi Z, Yin J, Jiang J, Guo L, He L, Chen Z, Tang Z, Qin S (2015). Comparative genome analysis of Prevotella intermedia strain isolated from infected root canal reveals features related to pathogenicity and adaptation. BMC Genomics.

[59] Faith JJ, Guruge JL, Charbonneau M, Subramanian S, Seedorf H, Goodman AL, Clemente JC, Knight R, Heath AC, Leibel RL, Rosenbaum M, Gordon JI (2013). The long-term stability of the human gut microbiota. Science.

[60] Koren O, Knights D, Gonzalez A, Waldron L, Segata N, Knight R, Huttenhower C, Ley RE (2013). A Guide to Enterotypes across the Human Body: Meta-Analysis of Microbial Community Structures in Human Microbiome Datasets. PLoS Comput Biol.

[61] Wang J, Linnenbrink M, Künzel S, Fernandes R, Nadeau MJ, Rosenstiel P, Baines JF (2014). Dietary history contributes to enterotype-like clustering and functional metagenomic content in the intestinal microbiome of wild mice. PNAS.

