# Erratum to: An integrative Bayesian Dirichlet-multinomial regression model for the analysis of taxonomic abundances in microbiome data

**DOI:** 10.1186/s12859-017-1606-z

**Published:** 2017-03-23

**Authors:** W. Duncan Wadsworth, Raffaele Argiento, Michele Guindani, Jessica Galloway-Pena, Samuel A. Shelburne, Marina Vannucci

**Affiliations:** 1 Department of Statistics, Rice University, Houston, TX USA; 2 ESOMAS Department, University of Torino and Collegio Carlo Alberto, Torino, Italy; 3 Department of Statistics, University of California, Irvine, CA USA; 4 Department of Infectious Disease, Infection Control, and Employee Health, The University of Texas MD Anderson Cancer Center, Houston, TX 77030 USA; 5 Department of Genomic Medicine, The University of Texas MD Anderson Cancer Center, Houston, TX 77030 USA

## Erratum

After publication of the original article [1] it was brought to our attention that author Samuel A. Shelburne was incorrectly included as Samuel A. Shelbourne. The correct spelling of the name is included in the author list of this erratum and has been updated in the original article.
