# Supplementary material for: An integrative Bayesian Dirichlet-multinomial regression model for the analysis of taxonomic abundances in microbiome data
Source: BMC Bioinformatics. 2017 Feb 8;18:94. doi: 10.1186/s12859-017-1516-0 (PMC5299727; doi:10.1186/s12859-017-1516-0)
Supplement: Additional file 1 — Comparison with other methods and sensitivity analysis in the simulation study. (PDF 142 kb) [file 12859_2017_1516_MOESM1_ESM.pdf]

Additional File

An Integrative Bayesian Dirichlet-Multinomial Regression Model for the Analysis of Taxonomic Abundances in Microbiome data

by W.D. Wadsworth, R. Argiento, M. Guindani, J. Galloway-Pena, S.A. Shelbourne, and M. Vannucci.

1. Simulation study: comparison with other methods

Figure **??** shows boxplots of the selection performance values over the 30 replicated datasets with dispersion parameter $\psi = 0.01$. Our proposed model either outperforms or is commensurate with the competing methods on all metrics.

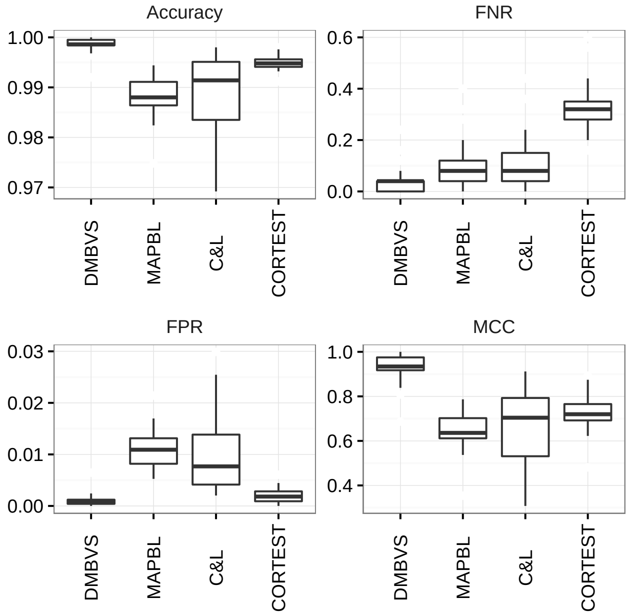

Figure 1: Simulated data: Comparison results of selection performances. DMBVS: Dirichlet–Multinomial Bayesian Variable Selection (our method), C&L: Chen and Li, MAPGL: Maximum A Posteriori Bayesian Lasso, CORTEST: Multiplicity Corrected Correlation Tests.

2. Simulation study: sensitivity analysis

We report the ROC curves obtained when investigating how sensitive the results are to varying values of the prior expected value of p_{pj}, i.e. $m \in \{0.005, 0.01, 0.05\}$ and the slab variance $r_{pj}^2 \in \{1, 10, 100\}$ in the variable selection procedure. The ROC curves complement the information provided by Table 2 in the main text.

$\psi = 0.1$

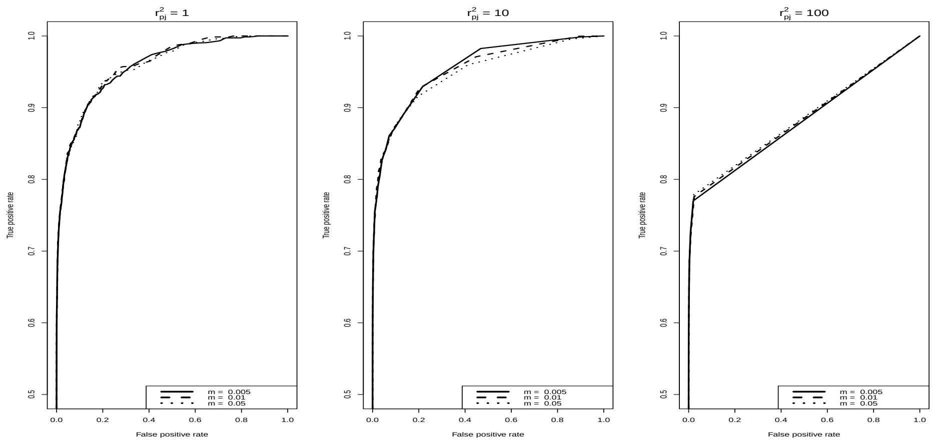

$\psi = 0.01$

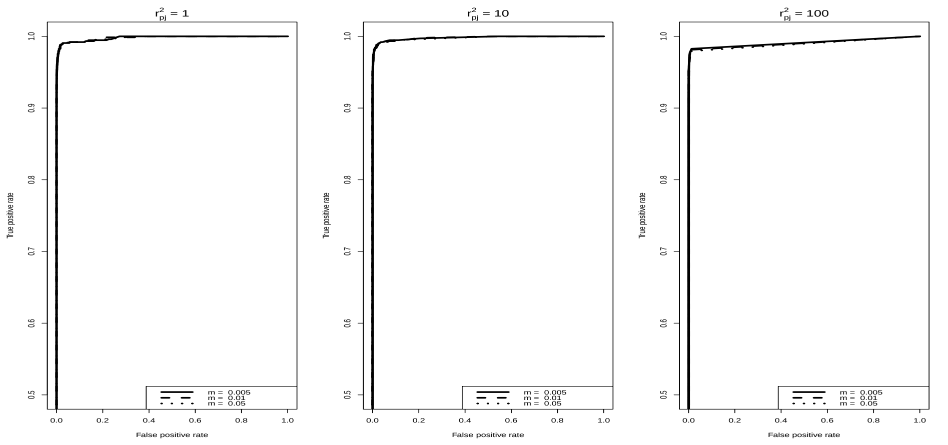

The results suggest that our procedure is quite robust to different specifications of relevant prior hyper-parameters.

In the Table below we evaluate the performance of our model for varying sample sizes $n = \{50, 100, 500\}$ and for varying values of the over-dispersion parameter $\psi = \{0.01, 0.1, 0.2\}$. Results are obtained with $r^2_{pj} = 10$ and $m = 0.01$ and supplement the information contained in Table 2 in the main text. Values are averages over 30 replicates. As expected, the results show that improved performance is achieved for larger sample sizes and decreasing overdispersion.

	$n = 50$	$n = 100$	$n = 500$
$\psi = 0.01$			
MCC	0.84	0.93	0.98
FPR	0.00	0.00	0.00
FNR	0.17	0.05	0.00
ACC	1.00	1.00	1.00
$\psi = 0.1$			
MCC	0.54	0.73	0.92
FPR	0.00	0.00	0.00
FNR	0.57	0.37	0.09
ACC	0.99	0.96	1.00
$\psi = 0.2$			
MCC	0.40	0.60	0.83
FPR	0.00	0.00	0.00
FNR	0.74	0.56	0.25
ACC	0.99	0.99	1.00
